# Predicting Emotional Valence of People Living with the Human Immunodeficiency Virus Using Daily Voice Clips: A Preliminary Study

**DOI:** 10.3390/healthcare9091148

**Published:** 2021-09-02

**Authors:** Ray F. Lin, Shu-Hsing Cheng, Yung-Ping Liu, Cheng-Pin Chen, Yi-Jyun Wang, Shu-Ying Chang

**Affiliations:** 1 Department of Industrial Engineering and Management, Yuan Ze University, Taoyuan 32003, Taiwan; juifeng@saturn.yzu.edu.tw (R.F.L.); puddingjyun1120@gmail.com (Y.-J.W.); 2 Department of Infectious Diseases, Taoyuan General Hospital, Ministry of Health and Welfare, Taoyuan 33004, Taiwan; shcheng@mail.tygh.gov.tw (S.-H.C.); cpc.y@nycu.edu.tw (C.-P.C.); ingrid45640@gmail.com (S.-Y.C.); 3 School of Public Health, Taipei Medical University, Taipei 110301, Taiwan; 4 Department of Industrial Engineering and Management, Chaoyang University of Technology, Taichung 413310, Taiwan; 5 Institute of Clinical Medicine, National Yang Ming Chiao Tung University, Taipei 11221, Taiwan

**Keywords:** HIV, speech emotion recognition, feature selection, artificial intelligence, clinical diagnosis

## Abstract

To detect depression in people living with the human immunodeficiency virus (PLHIV), this preliminary study developed an artificial intelligence (AI) model aimed at discriminating the emotional valence of PLHIV. Sixteen PLHIV recruited from the Taoyuan General Hospital, Ministry of Health and Welfare, participated in this study from 2019 to 2020. A self-developed mobile application (app) was installed on sixteen participants’ mobile phones and recorded their daily voice clips and emotional valence values. After data preprocessing of the collected voice clips was conducted, an open-source software, openSMILE, was applied to extract 384 voice features. These features were then tested with statistical methods to screen critical modeling features. Several decision-tree models were built based on various data combinations to test the effectiveness of feature selection methods. The developed model performed very well for individuals who reported an adequate amount of data with widely distributed valence values. The effectiveness of feature selection methods, limitations of collected data, and future research were discussed.

## 1. Introduction

### 1.1. PLHIV and Depression

Studies have found that people living with the human immunodeficiency virus (PLHIV) are more likely to be depressed than ordinary people due to negative emotions to initial diagnosis, the stress of living with chronic illness, perceived and internalized stigma, and the side effects of HIV drugs [1,2,3,4,5]. The prevalence of depression among PLHIV was between 18% and 81% [3,6,7,8]. Without proper interventions, depression in PLHIV can result in increased substance abuse [9,10], increased high-risk sexual behaviors [11], more rapid HIV progression [7,12], cognitive impairment [6,13,14], and increased risk of suicidality [5,15,16,17,18,19].

### 1.2. Depression Interventions for PLHIV

Recently, interventions that involve the use of consumer-grade hardware (i.e., mHealth [20]) were proposed to help PLHIV manage depression. For example, Swendeman et al. [21] designed a smartphone self-monitoring application to help PLHIV by asking PLHIV to fill surveys on medication adherence, mental health, substance use, and sexual risk behaviors, and brief text dairies on stressful events. van Luenen et al. [22] tested a Web-based cognitive behavioral therapy program to reduce PLHIV’s depressive symptoms and anxiety. Li et al. [23] studied a WeChat-based intervention to reduce the suicide rate of PLHIV. Although the abovementioned interventions were found helpful in improving mental health outcomes and decreasing suicidal risks in PLHIV, their successes rely on PLHIV’s willingness and adherence. Without PLHIV’s cooperation, these interventions had difficulties tracking PLHIV’s destructive emotions and preventing suicidal events effectively.

Integrating mHealth with artificial intelligence (AI) techniques could be a possible solution to track emotional states and prevent suicide. Schnall et al. [24] suggested that smartphones are a fantastic means to track people with chronic diseases. With sensing, computing, and data storage abilities, mobile devices could be an ideal tool to collect information from PLHIV, and the collected data can be tested for developing AI to track their emotional states. Among various data that mobile devices can collect, e.g., image, accelerometer, global positioning system, or temperature, voice can be easily collected from PLHIV in their verbal communications.

Studies have shown that emotion affects voice [24,25,26]. Many studies developed emotion recognition models using voice, e.g., [27,28,29,30]. However, there are limitations of these developed models for our use. First, most of these studies developed models to recognize categorical emotions, such as joy, guilt, contempt, anger, disgust, sadness, and fear [31,32,33]. While these models focus on the differences among these so-called “basic emotions,” they performed ineffectively in the degree of emotional valence. Second, the data used to develop these models were the databases, e.g., [34,35], that used acted speech simulated by actors. As Ayadi et al. [36] questioned, these databases’ naturalness is the biggest concern while using them for developing AI models. Lastly, the properties and the intentions of speak between these actors and PLHIV were different.

### 1.3. Research Objectives

While PLHIV have a high prevalence of depression and risks of suicidality, developing a means to track PLHIV’s emotional states in real-time may reduce unfortunate outcomes for PLHIV’s case managers or relatives prevent unfortunate events. In support of this hypothesis, this preliminary study aimed to test the integration of mHealth and AI to discriminate PLHIV’s emotions. Specific objectives were:

1. To collect PLHIV’s voice data using their mobile devices,
2. To screen critical voice features using statistic methods of correlation and analysis of variance (ANOVA),
3. To test AI modeling for discriminating PLHIV’s emotional valence and compare the effectiveness of the two statistical methods.

## 2. Material and Methods

### 2.1. Study Design and Setting

This study was conducted in the Taoyuan General Hospital, a 900-bed tertiary referred hospital located in Northern Taiwan. The hospital was designated to provide HIV care for more than 3500 PLHIV who resided in Taoyuan City [37]. To provide healthcare services and prevent unfortunate events of PLHIV, the Taoyuan General Hospital has been making efforts to track PLHIV’s emotional states. However, it is a considerable challenge for the HIV care team; the staff could merely investigate the emotional state through their regular visits per three months, restricted by the overloaded assignment. Hence, the hospital was seeking an effective means to overcome current limitations.

### 2.2. Participants and Sampling

Sixteen adult PLHIV, recruited from the Taoyuan General Hospital by convenience sampling, participated in this study from 2019 to 2020. The HIV care team recruited these participants from their PLHIV pool (approximately 3500 in total) via sending a flyer using Line and via in-person invitation in their clinic visits. Inclusion criteria for the current study were HIV-seropositive status, at least 18 years of age, and agreeing to participate in the study. Their demographic data are shown in Table 1 that summarizes information on age, sex, drug usage, sexual activity, HIV transmission, period of diagnosed HIV, anxiety, depression, and cellphone usage. Before participating in the study, their states of anxiety and depression were assessed using the Chinese version of the Hospital Anxiety and Depression Scale (HADS) [38].

Ethical approval was obtained from the Institutional Review Board of Taoyuan General Hospital. All the participants signed the written consent and knew their right to withdraw from the study if they wished before participating in the study.

### 2.3. Data Collection

**Figure 1:**
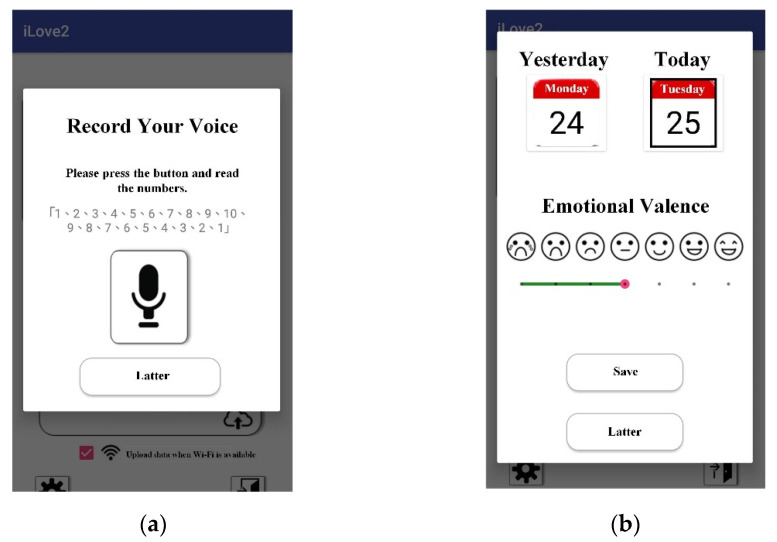
The self-developed iLove app. (**a**) Daily voice recording screenshot of iLove on which the participant record a daily voice clip while reading “1, 2, 3, 4, 5, 6, 7, 8, 9, 10, 9, 8, 7, 6, 5, 4, 3, 2, 1” in Chinese; (**b**) daily emotional valence reporting screenshot of iLove on which the participant reported their daily emotional valence by selecting responding facial expressions.

A self-developed mobile application (app), called iLove, was installed on the individuals’ mobile phones to record their daily voice clips and report daily emotional valence. As shown in Figure 1a, the participant used the mobile app to record a daily voice clip while reading “1, 2, 3, 4, 5, 6, 7, 8, 9, 10, 9, 8, 7, 6, 5, 4, 3, 2, 1” in Chinese. Voice clips of numerals were selected to eliminate unwanted data variances that result from spoken content. After each voice recording, participants reported their daily emotional valence by selecting responding facial expressions shown in Figure 1b. The seven facial expressions from left to right were recorded as values from one to seven. The collected data were sent to cloud storage at midnight and waited for further use.

### 2.4. Data Processing and Feature Screening

The downloaded voice data first went through data-preprocessing of reducing noises and cutting silent clips, and then an open-source software, openSMILE, IS09_emotion [39], was applied to extracted 384 voice features. As shown in Table 2, the software, openSMILE, automatically produced 384 voice features (i.e., a table grid indicates a feature) from a voice clip. These features were calculated based on 16 descriptors (see the leftmost column of Table 2), comprising root mean square (RMS) frame energy, zero crossings (ZCR), pitch frequency (F0), harmonics-to-noise ratio (HNR), and 12 mel-frequency cepstrum coefficients (MFCC) in accordance to HTK (Hidden Markov Model Toolkit)-based computation [40]. These 16 descriptors were used to capture de-differentiated (partial differential) values (*d’*) [35] to generate 16 non-personalized features [41]. Next, these 32 basic features were individually computed to obtain 12 statistical functionals, comprising mean, standard deviation (SD), skewness, kurtosis, maximum and minimum values, maximum and minimum positions, and range, as well as two linear regression coefficients (offset and slope) with their mean square error (MSE).

These 384 voice features were screened using two statistical methods: ANOVA and correlation. While using ANOVA, two *p*-values, 0.05 and 0.1, were set as criteria to screen two sets of critical features, whereas one *p*-value of 0.05 was set when using correlation.

### 2.5. Modeling

**Figure 2:**
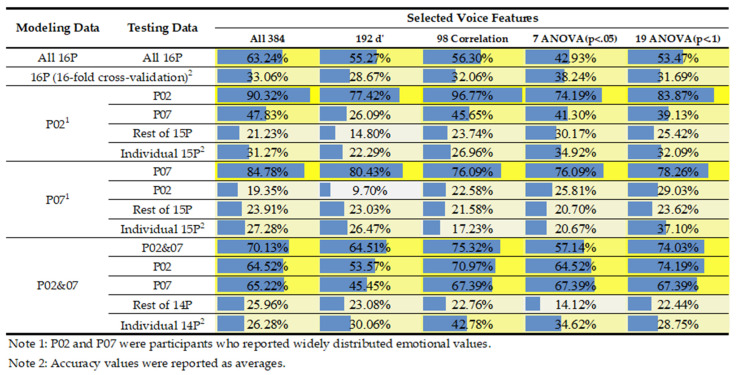
Comparison of modeling accuracy of using a variety of data set. A longer blue bar indicates a relatively higher accuracy rate, and a brighter yellow shade indicates a better modeling performance.

After extracting and screening voice features, the decision tree algorithm, tree.DecisionTree Regressor() through the scikit-learn library, was used to build various models based on different modeling data, testing data, and voice features. Because participants 2 and 7 were the only two participants who reported widely distributed emotional valence values, their data were specifically used when modeling and testing models. More explanations of the data will be detailed in the result section. As shown in Figure 2, four types of modeling data were used for modeling, comprising data of all 16 participants (All 16P), participant 2 (P02), participant 7 (P07), and participants 2 and 7 (P02&07). While developing models, the random state was set at 100, and the criterion was set as entropy. The max depth was tested with 4, 5, and 6, in which this range of values showed the best model performance. Only the best performance was reported in the result section.

The developed models predicted a variety of testing data. The model that used modeling data of 16P (Model_16P) tested the same data set of 16P (70% modeling and 30% testing). Sixteen-fold cross-validation (Models_16Folds) was performed using the data of 16P, in which 16 individual participant’s data were predicted by the models developed using the rest of 15 participant data in turns. The models that used modeling data of P02 (Model_P02) and P07 (Model_P07) tested the data of P02, P07, the other 15 participants as a whole (Rest of 15P), and the rest of 15 individual participants (Individual 15P). The model that used modeling data of P02&07 (Model_P02&07) tested the data of P02&07 (70% modeling, 30% testing), P02, P07, the other 14 participants as a whole (Rest of 14P), and the rest of 14 individual participants (Individual 14P).

To test the effectiveness of the two statistical methods for model development, five sets of voice features were considered, comprising all extracted features (All 384), 192 de-differentiated features (192 d’), correlation-screened features with *p* < 0.05, ANOVA-screened features with *p* < 0.05, and ANOVA-screened features with *p* < 0.1. While performing correlation and ANOVA, only the data of participants 2 and 7 were used because of their widely distributed emotional values.

## 3. Results

### 3.1. Collected Data

**Figure 3:**
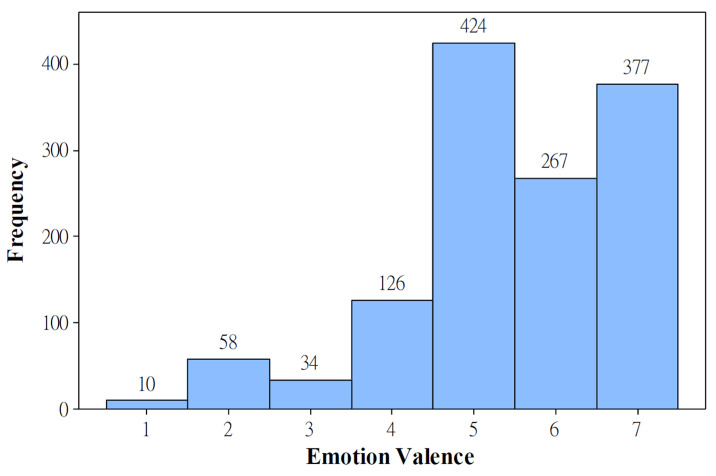
The distribution of data collection against the emotional valence of all 16 participants.

**Figure 4:**
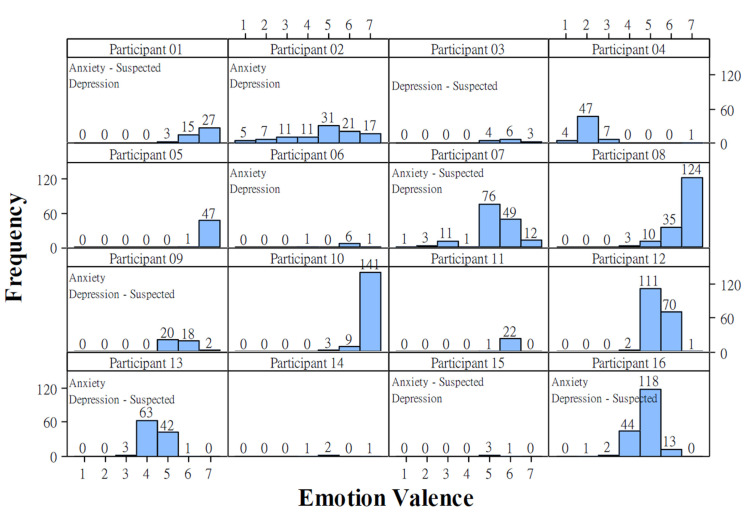
The distribution of data collection of 16 individual participants.

Sixteen participants reported 1296 successful data records, in which a participant recorded and uploaded both the voice clip and emotional valence in a day. However, as shown in Figure 3, the participants reported a large amount of high emotional valence. They reported happy states (three facial expressions on the right in Figure 1b) in 82.41% of days. Furthermore, individual differences existed. As shown in Figure 4, participants 1, 3, 5, 6, 8, 9, 10, 11, and 12 tended to report happy states, whereas participant 4 tended to report unhappy states. Participants 14 and 15 reported few successful data records. Participants 2, 7, 12, 13, and 16 reported relatively normal distributed emotions. However, participants 2 and 7 were the only two who reported widely distributed emotional valence values.

### 3.2. Critical Features Using Correlation and ANOVA

Table 2 shows critical features screened using correlation and ANOVA. There were 98 correlation-screened features (indicated with the yellow shading), seven ANOVA-screened features with *p* < 0.05 (indicated with *), and 19 ANOVA-screened features with *p* < 0.1 (indicated with ^ and *). The table also shows all the 384 *d* features (all the combinations) and 192 *d’* features (the half combinations on the right side). These five sets of features were used to develop models.

### 3.3. Model Performance

**Figure 5:**
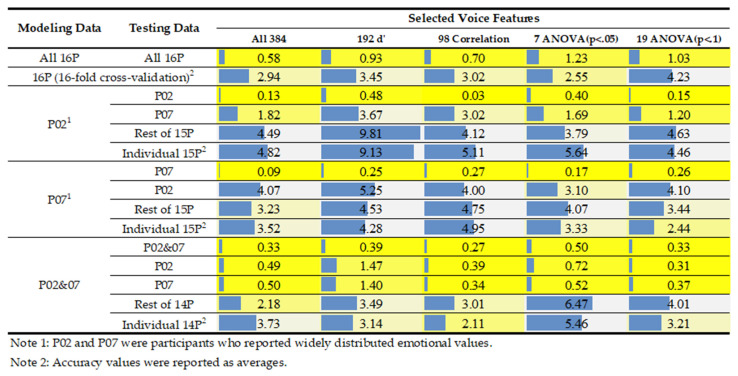
The comparison of modeling MSE of various data set. A longer blue bar indicates a relatively higher value, and a brighter yellow shade indicates a better modeling performance.

The accuracy and mean squared error (MSE) of developed models are shown in Figure 2 and Figure 5, respectively. First, regarding the use of all participants’ data, Model_16P that used all 384 voice features obtained the best performance (63.24% accuracy rate and 0.58 MSE) compared to the models that used the other four feature sets (i.e., 192 *d’* features, 98 correlation-screened features, seven ANOVA-screened features, and 19 ANOVA-screened features). However, this is an overfitting result because the performance decreased dramatically (approximately 30%) when doing the 16-fold cross-validation. The results of 16-fold cross-validation showed that using 7 ANOVA-screened features resulted in the best performance (38.24% accuracy rate and 2.55 MSE).

**Figure 6:**
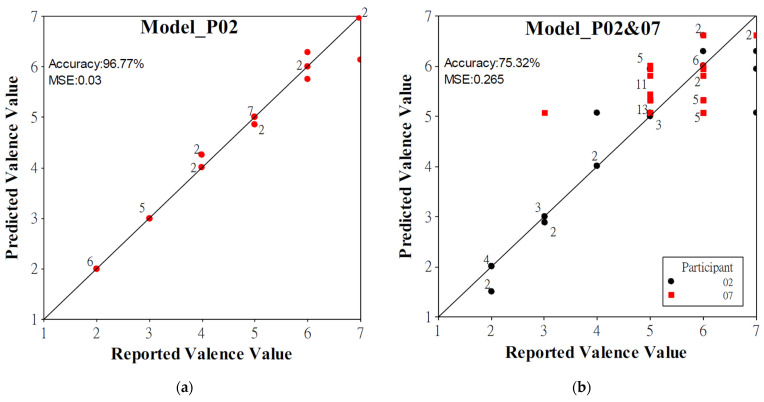
Visualization of model performance. (**a**) Performance of Model_P02 using 98 correlation-screened features (70% modeling, 30% testing); (**b**) performance of Model_P02&07 using 98 correlation-screened features (70% modeling, 30% testing). The number on the symbols represents the number of data with the same predictions.

Second, while using individual modeling data of P02 and P07, Model_P02 and Model_P07 had excellent performance. When predicting their own data sets, Model_P02 using 98 correlation-screened features had the best performance (96.77% accuracy rate and 0.03 MSE, see Figure 6a for a visual representation), whereas Model_P07 using the data of All 384 features had the best performance (84.78% accuracy rate and 0.09 MSE). When the two models predicted each other’s data, Model_P02 using the data of All 384 features had the best performance to predict the data of P07 (47.83% accuracy rate and 1.82 MSE), whereas Model_P07 using 19 ANOVA features had the best performance to predict the data of P02 (29.03% accuracy rate and 4.1 MSE). When the data of the rest of 15 participants were tested, Model_P02 using 7 ANOVA-screened features obtained the best performance for predicting Rest of 15P (30.17% accuracy rate and 3.79 MSE) and Individual 15P (34.92% accuracy rate and 5.64 MSE), whereas Model_P07 obtained the best performance for predicting Rest of 15P (23.91% accuracy rate and 3.23 MSE) using All 384 features and Individual 15P (37.1% accuracy rate and 2.44 MSE) using 19 ANOVA-screened features.

Finally, while developing models using combined data of participants 2 and 7, Model_P02&07 using 98 correlation-screened features obtained the best performance for predicting the combined data sets (75.32% accuracy rate and 0.27 MSE, see Figure 6b for a visual representation). Compared to Model_P02 and Model_P07, Model_P02&07 had a much better model performance for predicting the testing data of P02, P07, Rest of 14P, and Individual 14P, although Model_P02&07 cannot compete with Model_P02 and Model_P07 when predicting their own data sets. Model_P02&07 had the best performance of the testing data of P02 (74.19% accuracy rate and 0.31 MSE) using 19 ANOVA-screened features, of P07 (67.39% accuracy rate and 0.34 MSE) using 98 correlation-screened features, of Rest of 14P (25.96% accuracy rate and 2.18 MSE) using all 384 features, and of Individual 14P (42.78% accuracy rate and 2.11 MSE) using 98 correlation-screened features.

### 3.4. Decision Tree

**Figure 7:**
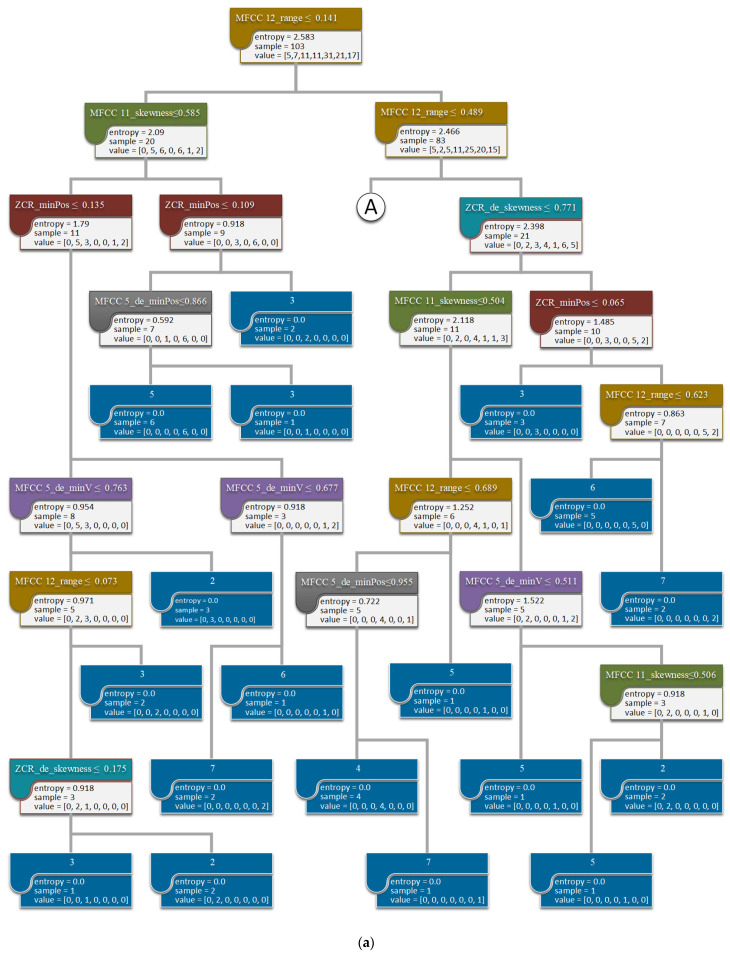

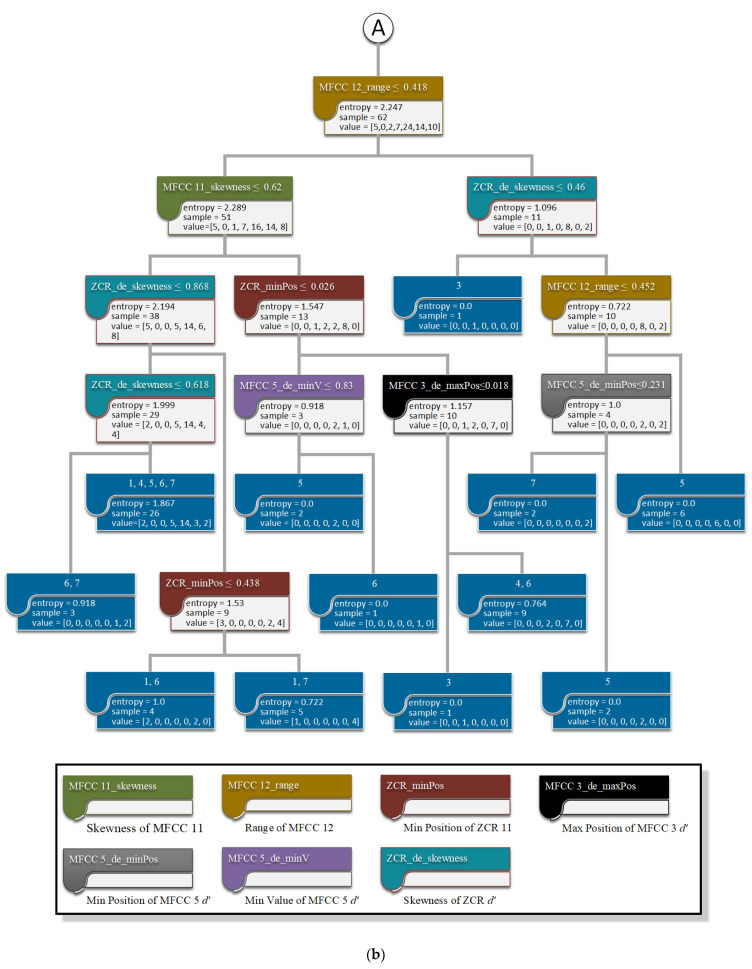
Visualization of the developed decision tree of Model_P02 using 7 ANOVA-screened features. (**a**) Part one of Figure 7; (**b**) Part two of Figure 7.

All the developed models shown in Figure 2 could be visualized as trees showing decision rules. Due to limited space, only the decision tree of Model_P02 using 7 ANOVA-screened features is detailed here as an example. As shown in Figure 7a, the decision tree shows how the model determines the emotional valence value by using 30 decision rules. These rules are the paths from the first judgment node through the following judgment nodes (seven colored nodes indicate the seven critical features screened using ANOVA) to all the 30 end nodes (represented as solid blue ones). For example, the rightmost end node in the last level (Figure 7b) shows the prediction made with a rule, IF MFCC 12_range > 0.141 AND MFCC 12_range ≤ 0.489 AND MFCC 12_range ≤ 0.418 AND ZCR_de_skewness > 0.46 AND MFCC 12_range ≤ 0.452 AND MFCC 5_de_minPos > 0.231 THEN label = 5. This end node also shows two samples that matched the rule and had the assigned prediction of 5. Based on these decision rules, MFCC 12_range is the most critical feature to influence emotional valence. The feature was used as the first judgment node for all the 30 rules and applied the most frequently in the following decisions.

## 4. Discussion

### 4.1. Limitations of Collected Data

Although it took nearly two years to collect data from 16 PLHIV, the data collection was not ideal. As shown in Figure 3, the collected emotional valence data concentrated on only a few high valence values. The unbalanced data would make the developed model tend to predict the valence as the values with great numbers of data (i.e., 3, 6, and 7). Furthermore, as shown in Figure 4, only two participants (i.e., participants 2 and 7) reported widely distributed valence values. The narrow ranges of emotional valence values reported by the other participants (participants 1, 3, 4, 5, 6, 9, 11, and 15) would result in an overfitting issue when applying all participants’ data for developing AI models. That is, the model tended to recognize emotional valence using features related to individuals instead of using the features with emotional properties. The performance comparison of Model_P16 and Models_16Folds confirmed this overfitting issue. The modeling performance dramatically decreased when predicting the emotional valence of an additional patient whose data were not used in modeling. Furthermore, other than the limited amount of collected data, we should question the accuracy of emotional valence values reported by the participants. As shown in Figure 4, there were some participants (i.e., 5, 8, and 10) who consistently reported extremely high emotional valence.

Collecting sufficient and valid data from PLHIV for an extended period is challenging. Although the mobile app automatically showed a reminder to the participants every day, the participants could ignore the reminder or report unsuccessful information due to various reasons that include being busy, feeling troublesome, and forgetting. Strategies, such as providing monetary incentives and monitoring reported data daily by an experimenter, should increase the motivation and data validation for future data collections.

### 4.2. Model Performance When Using the Modeling Data of Participants 2 and 7

To overcome the limitations of collected data, we tested the data of participants 2 and 7 for developing models. As shown in Figure 2 and Figure 5, Model_P02 and Model_P07, which used merely a single participant’s data for modeling, could compete with Models_16Folds that used 15 participants’ data when predicting the other participants’ emotions. Furthermore, Model_P02&07 that used the two participants’ data performed much better than Models_16Folds did when 98 correlation-screened features were used (42.78% vs. 32.06% accuracy rate and 2.11 vs. 3.02 MSE). Hence, widely distributed emotional valence data are critical for developing effective models for practical use.

It was surprising that Model_P02 and Model_P07 had excellent modeling performance when modeling data and testing data are from a single participant. As shown in Figure 6, the two models obtained very high accuracy rates and ideally predicted low emotional valence values. The most remarkable performance (96.77% accuracy rate and 0.03 MSE) was obtained by Model_P02 using 98 correlation-screened features. Compared to participant 7, participant 2 reported relatively wide and even valence values data (Figure 4). Hence, if a participant reported an adequate amount of widely distributed emotional values, a high-performance AI model could be expected for detecting his emotional valence.

### 4.3. Comparisons of Screened Voice Features

Except for manipulating modeling data, another method we used to overcome the overfitting issue was applying different voice features. As abovementioned, we stated that using all 384 features provided by openSMILE could result in the issue that the model applied the voice features related to individual differences instead of emotion-related features. Hence, the *d’* features and two statistical methods were applied to screen critical features. The *d’* features that use the derivative-based method [35] were proposed to produce non-personalized emotional characteristics and hence reduce individual differences [41]. Correlation and ANOVA tested the statistical relationships between emotional valence and voice features. According to our results, the *d’* features did not show their superiority in modeling over the two statistical methods. Perhaps one reason was that all participants were male, and they had fewer individual differences in voice. As shown in Table 2, the numbers of features screened by correlation, ANOVA with *p* < 0.1, and ANOVA with *p* < 0.05 were 98, 19, and 7, respectively. Although we did not have consistent results to conclude the best method for screening voice features yet, we see the benefits of using statistical methods to reduce the number of voice features effectively. Most importantly, the statistical method’s application can overcome the overfitting issue using all the 384 *d* features for modeling. For example, using ANOVA features (either the numbers of 7 or 19), Model_P02 had better performance for predicting the other participants’ emotional valence (both individuals and the group). Using 19 ANOVA features, Model_P07 had better performance for predicting the other individual participants’ emotional valence. Using any of the three statistical features, Model_P02&07 had better performance for predicting the other individual participants’ emotional valence.

### 4.4. Contribution and Future Research

While mHealth [20,21,22,23] has been promoted to help PLHIV manage depression, this preliminary study attempts to integrate mHealth with AI to track PLHIV’s emotional valence. We show the potential to develop a low-emotional-valence detection model and statistical methods to use the limited data with currently available data efficiently. Due to the data limitations, we cannot develop an effective AI model for detecting PLHIV’s low emotional valence yet. However, we have demonstrated that a general model (i.e., Model_P02&07) could be developed with widely distributed emotional data to provide reasonable performance. Additionally, excellent models (i.e., Model_P02 and Model_P07) could be developed for individuals if they provided widely distributed emotional data. Hence, our future research is to collect more data from participants, especially the participants who can report widely distributed emotional data. However, we do not expect that the data will increase dramatically due to the limited number of participants and the difficulties of reporting data every day. Therefore, the use of statistical methods is necessary to screen critical and meaningful voice features. With more data available, other AI methods could be tested while modeling. This study tested support vector machine, random forest, anomaly detection, and decision tree and found the decision tree was superior to the other three. Due to the characteristic of our collected data (fewer low emotional valence values), the method of anomaly detection may be suitable for developing models when more data are available. Besides testing these methods, the subsequent study is to develop a general model first and install this general model in a new PLHIV to start practical use. After a while, a transfer learning technique can use feedback to adjust the model for personal use.

Once data collection limitations are overcome, our ultimate goal is to develop an effective AI model installed in a PLHIV if he/she agrees. The AI model is expected to detect bad emotional states, and the participant’s cellphone will automatically send a message to his/her case manager. The case manager then provides immediate care to the patient and hence reduces the unfortunate event.

## 5. Conclusions

This preliminary study shows the potential of using daily voice clips collected using a smartphone to develop an AI model for detecting the negative emotional states of PLHIV. With the collected data of the two specific participants, the developed decision-tree models predicted their emotional valence measured in a seven-scale range. The applications of correlation and ANOVA help screen voice features for developing AI models. However, these interpretations are limited due to the number of participants who reported adequate data. The following research is continuing to collect valuable data from more participants. While collecting data, emotional valence data reported by the participants need to be adequate and, most importantly, widely distributed on the emotional valence scale. The ultimate goal of this data collection is to improve the AI model detection of depression for case managers to effectively provide treatment and improve the mental health outcomes of PLHIV.

## Figures and Tables

**Table 1:** Demographic data of the 16 participants.

Characteristics	*n*	%
**Sample size**	16	100
**Age**		
Mean (years)	34.53	―
SD (years)	5.72	―
**Sex**		
Male	16	100
**Occupational State**		
Employed (stable)	6	37.5
Employed (unstable)	6	37.5
Self-employed	1	6.25
Unemployed	3	18.75
**Drugs even taken**		
Amphetamine	7	43.75
Gamma-hydroxybutyrate	3	18.75
Rush	2	12.5
Took within 3 months	7	43.75
Never took	9	56.25
**Period between diagnosis of HIV infection**		
Mean (years)	7.87	―
SD (years)	4.26	―
**Anxiety**		
Definite (score 11–21)	5	31.25
Doubtful (score 8–10)	3	18.75
No (score 07)	8	50
Mean (score)	7.63	―
SD (score)	5.91	―
**Depression**		
Definite (score 11–21)	5	31.25
Doubtful (score 8–10)	4	25
No (score 0–7)	7	43.75
Mean (score)	7.69	―
SD (score)	4.17	―

**Table 2:** Screened voice features using ANOVA and correlation from 384 voice features produced by openSMILE.

Effects		Original Descriptors (*d*)												Delta Regression Coefficients of Descriptors (*d’*)											
		Mean	SD	Skewness	Kurtosis	Max Value	Min Value	Max Position	Min Position	Range	Offset	Slope	MSE	Mean	SD	Skewness	Kurtosis	Max Value	Min Value	Max Position	Min Position	Range	Offset	Slope	MSE
RMS									^														^		
ZCR						^	^	^	*							*									
F0																									
HNR								^																	
MFCC	1																				^				
	2																							^	
	3																			*					
	4							^																	
	5																		*		*				
	6															^				^					
	7																								
	8						^																		
	9																								
	10																								
	11			*																					
	12									*															

^ indicates *p* < 0.1 using ANOVA; * indicates *p* < 0.05 using ANOVA; a yellow shade indicates *p* < 0.05 using correlation; SD = Stadnard Deviation; MSE = Mean Square Error; RMS = Root Mean Square; ZCR = Zero Crossings; F0 = Pitch Frequency; HNR = Harmonics-To-Noise Ratio; MFCC = mel-frequency cepstrum coefficients.
